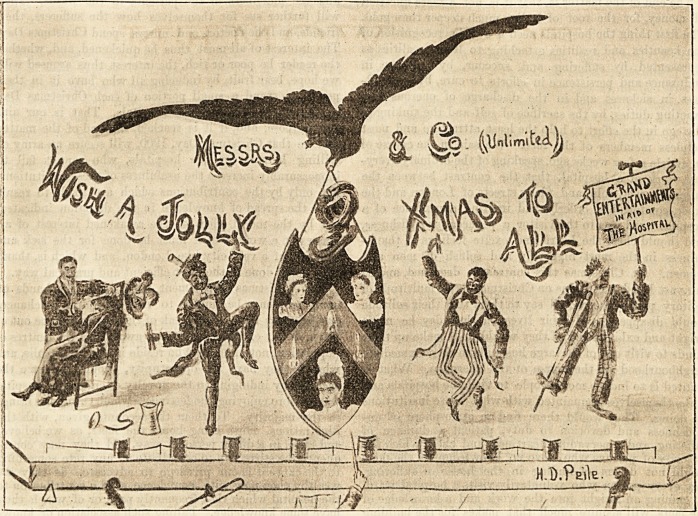# Our Christmas Supplement

**Published:** 1900-12-15

**Authors:** 


### OUR CHRISTMAS SUPPLEMENT.

### THE END OF THE MATTER.

In all lives day follows day, and each day's end leaves us with the feeling that the next day's work and responsibilities will begin with the morning light. So our Christmas Number, which has now been published for many consecutive years in the cause of the hospitals, is for the present year but the beginning of a new day, and thus we commence this year with the End of the Matter. What is that end but the responsibilities of every intelligent resident in London and the home counties towards the hospitals, and the necessity, which at the moment is intensified by the circumstances of the year about to close, to adequately support these institutions? When we commenced our work for the hospitals thirty years ago the public at large took very little interest in them. They were at that time regarded with anything but universal favour, and were surrounded with an atmosphere of suspicion, due mainly to the difficulties which beset the treatment of a large number of sick people in great buildings devoted to the purpose. The medical profession differs from all other professions in the fact that, speaking broadly, every member of it is imbued with the sense of his individual responsibility to continuously search out and apply the most suitable remedy for every ailment which attacks the human body. To this circumstance is due the immense progress which has been made in sanitation, in methods of treatment, in skilled nursing, in the closest attention to every detail which can increase the comfort or expedite the recovery of the sufferers who enter the hospitals. The facts are so patent and the results so admirable that to-day the public at large realises that for everybody, whatever their wealth or position, when overtaken by serious illness there is no place like a hospital.

In such circumstances it should be a work of supererogation to advocate the cause of the hospitals with all classes of the community. If we could put the clock back, and restrict the numbers receiving treatment at the hospitals to a total equivalent to that of twenty years ago, the funds now given would no doubt be sufficient to place the hospitals in a sound financial position. The causes which underlie this result may be traced without difficulty. As the percentage of recoveries in hospitals has steadily increased, and the percentage of deaths has steadily diminished, the popularity of the hospitals has risen to so high a pitch, and the confidence in the hospitals is so universal, that no one, not even that arch-anti-vivisectionist and untiring foe of hospital managers, Mr. Stephen Coleridge, ventures to pursue his pro-animal, anti-human *rôle*, without ending with a declaration to the effect that his aim "is not to attack the hospitals, but to defend them." The army of givers every year increases, but every year the population of London increases in greater proportion, whilst death in reaping its victims sadly thins the ranks of the generous, leaving places to be filled, and new and willing hands to be found to replenish the coffers of our hospitals. So the army of givers needs recruits year by year in increasing numbers from every rank and station of life, from the plutocrat to the peasant.

The end of the matter is, that it is not a mere question of money, for the root of it lies much deeper than gold. The first thing the hospitals need is a wider recognition of the beauties and realities attaching to living realities as represented by suffering and succour, by patience in endurance and persistence in efforts to cure, by cheerfulness in sickness and in the discharge of onerous and exacting duties, by the sacrifice of self and the sinking of the ego in the effort to help the least attractive and most helpless members of the race. We said in the course of an article a few weeks ago, speaking of the ordinary everyday work of a hospital, that the contrast between the miserably wet, cold, and filthy streets of London and the quiet, comfortable, orderly, and inviting appearance of a hospital ward was to the visitor a panacea for selfishness, and should cheer the heart and excite feelings of thankfulness in the most niggardly and selfish of men and women. At Christmas this contrast is deepened, and as our vast London contains on Christmas Day a multitude of solitary people, we would say to them that their solitude would disappear, and their lives on that day be made bright and exhilarating, if they would only make up their minds to visit one of the large hospitals in their immediate neighbourhood in the course of the afternoon. What is wanted is to induce more people to visit the hospitals and make themselves acquainted with what these institutions are doing. They would there find an atmosphere of unselfishness and devotion to duty, patient endurance of suffering, and a pervading heartiness and cheer which the majority of persons who have no knowledge of the facts would not dream of finding in the house of suffering. Yet every person who voluntarily visits a hospital ward, by gaining an insight into the work and a knowledge of the facts, will acquire much that makes for the building up of individual character whereby they may hope to become convinced voluntary supporters of these noble institutions, which are the glory of the Empire and the envy of the world.

The sceptics may smile, but everything we have written is demonstrably provable, and we challenge them to put our statements to the test by a visit to the hospitals. As a preparation for their visit we invite a careful perusal of the pages which follow, in which Christmas is treated from the point of view of the inner life of the wealthy, the artisan, the slum-dweller at home in London on Christmas Day. The reader, as he turns over the pages, will learn with interest how Christmas is spent in the hospitals, what the preparations for a merry Christmas in a hospital mean, the efforts which are made to lighten suffering, to diminish the effects of separation from friends, and the loneliness of the patients in the wards. They will further see for themselves how the sufferers, their friends, and the doctors and nurses spend Christmas Day. The interest of all must thus be quickened, and, whether the reader be poor or rich, the interest thus aroused will, we hope, bear fruit, by inducing all who have it in their power to spend a small portion of each Christmas Day in the wards of one of the hospitals. That is our aim and purpose, and, if it is reached, the end of the matter will be that Christmas Day, 1900, will secure an army of willing helpers for the hospitals, who cannot fail to immeasurably increase the usefulness of these institutions, not only by the contributions which are likely to result from the spread of knowledge in the direction indicated, but by the moral effect of the awakened interest of all classes in a work which must be done for the sick and suffering of a vast city like London, and which is, thank God, being done in the most efficient and practical way.

This Christmas Supplement must reach the hands of many who may be unable to visit a hospital, perchance, on Christmas Day. We speak of those who may be out of town, or out of the country, or away from the centres of population, not of those who reside in the Metropolis, and who, if they have the opportunity, should welcome the occasion by indulging in the novelty of entering a hospital ward before enjoying the festivities which Christmas brings to the majority. To them the accounts given, with the illustrations, must bring home the facts, as we believe, and hope, in a direct personal way, and they may consequently be stirred up to contribute at any rate something to the cause it is our privilege to advocate. If they are wealthy they may with confidence send a contribution to the hospital which they frequently pass, or of which they have some knowledge. Failing such association or knowledge, the wealthy may thankfully send a generous gift for the hospitals to the Prince of Wales's Hospital Fund, Bank of England, London, E.C.; whilst those who are not wealthy, or whose means are limited, can rest assured that their shillings and half-crowns, and better still their personal service, will be welcomed, if sent to or left with the Honorary Secretaries of the League of Mercy, 29 Southampton Street, Strand, London, W.C. Thus it will be seen the end of the matter is to give of oneself, and to render practical help to a cause which appeals to all thoughtful persons independent of creed, race, or sex. To all we would say, Visit a hospital if you can; but in any case stay not upon the order of your GIVING, but GIVE at ONCE!

### SWEET CHARITY'S GUIDE TO CHRISTMAS GIVERS.

### GENERAL HOSPITALS.

**Charing Cross Hospital,** Agar Street, West Strand, W.C.—This institution is situated in the heart of the Metropolis, and in some of its most densely-populated and poor districts. Surrounded by crowded thoroughfares, it has to provide for an unusual number of accidents. The hospital has a convalescent home, containing 50 beds, situated on Limpsfield Common. £15,000 a year is required annually from voluntary sources to maintain the hospital and the home. Secretary, Mr. A. E. Reade.

**Great Northern Central Hospital,** Holloway Road, N.—Letters of recommendation are not required at this hospital; 159 beds are now open. Over 2,000 in-patients are treated annually. The hospital is unendowed, and the reliable income is quite inadequate to meet the necessary expenditure, over £10,000 being required annually from voluntary sources. The receipts of the hospital have been adversely affected this year by the competition of the war funds, and a deficit of £3,000 is probable in consequence. Special precautions are taken here to restrict the benefits of the charity to the sick poor. Secretary, Mr. Lewis H. Glenton-Kerr.

**Guy's Hospital,** London Bridge, S.E.—At the present time Guy's Hospital is in urgent need of substantial support from the benevolent in order to bring it to the highest state of efficiency and usefulness, and £25,000 per annum must be obtained from voluntary sources to supply the deficiency in the income necessary for the maintenance of the complete establishment. Treasurer, Mr. H. Cosmo O. Bonsor; Superintendent, Dr. E. C. Perry.

**Hampstead Hospital,** Parliament Hill, N.W.—During 1899 there were 385 in-patients besides 600 minor accidents and casualties treated in the hospital. There were also 2,620 attendances in the out-patient department. It is estimated that the expenditure in 1900 will considerably exceed the income. Additional funds are urgently needed to meet this deficiency. The present hospital being totally inade-

### Christmas in the Home.

### THE WEALTHY HOME.

### THE FAIRY GODMOTHER.

THERE was no doubt about the wealth. The staircase was of solid marble, the carpets were soft as velvet, the dishes were of costly porcelain or of glittering silver, and the servants had meat three times a day. Moreover, Robson, the butler, rarely deigned to put coals on the fires himself; he rang for John, the footman, to perform the duty. This fact alone spoke volumes as to the manner in which the establishment was conducted.

It was Christmas Eve. Below stairs all were very busy. Nearly all were very cross. How could they be otherwise when there was so much to do?

Mrs. Blackman, the cook, was growling audibly.

"Talk about a happy Christmas, indeed! Precious little o' that sort o' thing we're likely to get with eighteen to dinner to-morrow. Work! work! work! all days alike, and no consideration for the servants, of course not!"

"But," ventured the kitchenmaid, who was new to the situation, "don't the fam'ly give any presents?"

"Why, for certain they do," interrupted the footman, "'cos the haristocracy would be ashamed not to. You'll git your suv'reign right enough, and the others tips in proportion. But, Lor', that ain't much to speak of!"

And Eliza the scullery-maid, wondering if she were too low in the scale to be remembered, listened with all her ears, and said nothing.

Upstairs Lady Money reclined in an armchair, in her morning gown of Paris make, and languidly gave orders to her housekeeper. The ménu of the morrow's feast had been submitted to her, and she had approved, primarily, apparently, because it was less trouble than finding fault. But she had been seriously annoyed by the florist sending a note to announce his regret that, owing to the inclemency of the season, he had been unable to procure sufficient pink roses for the scheme of table decoration which her ladyship had selected.

"Really, these people are incorrigible. It is bad enough to have to get through Christmas at all, without the tradesmen failing in this annoying fashion. I do hope you will see that the carpenters get the wreaths in their places before Sir Francis returns from town. Nothing annoys him more than to find the place upside down upon these occasions. I begin to regret that we did not after all go to Brighton for Christmas. That will do, thank you, and please send Miss Gwen to me," and the beautiful woman with the tired face lay back and awaited the coming of her child.

In a few minutes the door opened. A sweet-looking little maiden, whose fashionable and expensive dress failed to detract from her childish grace, came bounding in, and as she flung her arms around her mother's neck the discontent vanished, and love and happiness for the moment reigned supreme.

"I am going out, darling. Would you like to come too?"

"Yes, mother. Aren't you glad it's Christmas Eve? I am. The shops will look so bright, and it's nice to see the people looking so happy. But, mother," and the little face suddenly clouded, "every one isn't happy, even on Christmas Eve. Eliza isn't."

Lady Money laughed. "My dear child, what do you know about Eliza?"

Gwen looked wise. "I know a great deal, and I'll tell you how. I coaxed Nurse to let me go down into the kitchen to stir the Christmas puddings."

Lady Money looked vexed. "Nurse should have had the puddings brought up to you, dear."

"Not a bit, mother; that would have been all wrong. In all my story books the children always go down and stir along with everybody else. When I had done stirring I saw Eliza standing near, and I remembered Nurse had said that she had a little brother about as big as me in the hospital; so I just asked her how he was, and she said he was better, and had come out, and then she began to cry. So I said, 'Why, Eliza, you are funny to cry because he's come out. I should have thought you'd have been glad he's getting better,' and she said she was, but he had so much wanted to stop in St. Faith's till after Christmas Day, because he'd have had presents and such good things to eat, and now he won't have any because they're so poor. Is that true, mother?"

---

### SWEET CHARITY'S GUIDE TO CHRISTMAS GIVERS.

### GENERAL HOSPITALS—*Continued.*

quate to meet the wants of the neighbourhood, a building fund has been started for the erection of a new hospital. Hon. Secretary, Mr. R. A. Owthwaite.

**Italian Hospital,** Queen Square, Bloomsbury, W.C.—New buildings were opened in March last for 50 in-patients, and a large out-patient department. The hospital is a general one, free to the sick and injured poor of all nationalities and creeds, and entirely dependent on voluntary support. Nearly 40 per cent. of the patients being British subjects, the committee believe they will not appeal in vain to the benevolent in this country for a part of the help needed, especially when the long-standing and cordial friendship between England and Italy is remembered. Some of the beds at present remain unoccupied for lack of funds, but with a comparatively small addition to its income the hospital's full benefits (which are greatly needed) would be available. Hon. Secretary, Cav. Luciano de Rin.

**King's College Hospital,** Portugal Street, Lincoln's Inn, W.C.—2,408 in and about 20,000 out-patients were treated in 1899, and the expenditure exceeded the income by nearly £6,000. Warden, Rev. N. Bromley.

**London Homœopathic Hospital,** Great Ormond Street, W.C. This hospital has completed its first half-century. Its in-patient number 1,100 annually. It gives 35,000 out-patient consultations every year. The annual expenditure at this hospital is at the rate of £10,000. The reliable income is about £7,000 per annum. There is, therefore, an urgent need of an immediate and substantial increase in the annual subscription list. Treasurer, Lord Cawdor, 7 Princes Gardens, S.W. Secretary-superintendent, Mr. G. A. Cross.

**London Hospital,** Whitechapel, E.—This, the largest voluntary hospital in this country, stands in greater need of help than ever. Important and much-needed alterations

"I don't know, dear. Perhaps it is. I really never asked about Eliza's people."

"Then, mother, I was thinking it would be so nice if I might be a fairy godmother just for once. I know I'm not really old enough, for fairy godmothers ought to have grey hair and walk with a stick; but I'm sure I've read of little girls being fairy godmothers sometimes, and it would be lovely!"

"Well, darling, you shall do anything that will make you happy. Now tell me this wonderful scheme."

Gwen went off into a ripple of laughter.

"Oh, mother, it isn't as big as a 'scheme,' and I've only though of a little, and you must help me with the rest. But what I should like would be for the little boy somehow or another to get all the things which he wanted so badly, and not even to know where they came from. You see I always get heaps of presents, and I've plenty of money in my money-box, so you won't mind my spending some, will you?" And it would have needed a hard heart to resist the pleading face.

The first result of the scheming was that Robson was requested to summon Eliza to her mistress' boudoir. Poor child! she could think of nothing but instant dismissal which would necessitate so unusual a proceeding, and such was her terror that the hearts of her fellow servants were touched by her distress, and she was bidden to "Cheer up a bit, for even if she did 'get the sack' she shouldn't go home empty-handed. They'd send round the hat in the kitchen and the housekeeper's room, and she should take home a Christmas box with her." But it was a very frightened little face which appeared upstairs, and when Lady Money commenced to ask her a great many qnestions about her widowed mother and the illness of her brother Willie and did not scold her, the terror gave way to mystification. A comforting side of the interview was that Miss Gwen was sitting by, smiling all the time; but still that did not account for the strangeness of her mistress writing down the address of her scullery maid's modest home in an obscure London street. Ultimately, she was dismissed with, "You may go now, Eliza," and, almost before she knew, the words had slipped out:

"Then you're not going to send me away, my lady."

Eliza had seldom been in her mistress' presence, but she was sure she had never seen her smile so pleasantly as she did this Christmas Eve as she replied, "No, of course not. It is Miss Gwen who is going to send something else away."

And then a wonderful thing happened. Miss Gwen came down into the kitchen with a message to Mrs. Blackman from her mother that she wished a hamper packing up to be ready directly after lunch, and said the child clapping her hands with glee, "You are to leave room for a turkey to be put in from the shop, but please pack in everything else which a little boy as big as me, who has been ill, would like on Christmas day, and you mustn't forget the plum pudding."

Then a hamper was packed so full that there was hardly room for the turkey, and everybody downstairs was, if possible, busier than before, but somehow the true spirit of Christmas seemed to have entered with little Gwen for the grumbling had ceased, and as John was given the basket to carry up to the front door, the cook was heard to remark, "Well! wonders will never cease. I've been here over three year, and it's the first hamper as I've ever packed. But it makes you feel a bit more like Christmas, that it do!"

That afternoon a carriage with a couple of splendid horses, a coachman and footman cosy in large fur capes, and a lady with a fair-haired child pulled up at No. 19 Smith Street, the same house as that into which two days before a little boy had been carried from a cab. When the gorgeous footman, after some knocking, had brought a tidy-looking woman in a widow's cap to the door, he returned to the carriage and a small white clad figure sprang out, followed by the footman laden with parcels. "Please," said the childish voice, "will you give the hamper to Willie, and the box of toys and books, and the eiderdown, and tell him they came from a fairy godmother?" And not waiting for the thanks which the astonished mother found it difficult to express, with a merry laugh the messenger of happiness ran back to the carriage, and was soon lost to view.

In the letter-box of St. Faith's Hospital next morning there were two letters. One enclosed a cheque for £50 "from a mother whose little girl loves the hospital children to be happy," the other contained a shilling and sixpennyworth of coppers, and in an illiterate hand were these words:

"Willie's dinner, wot he don't want, 'cos a fairy godmother brought him lots of things."

### SWEET CHARITY'S GUIDE TO CHRISTMAS GIVERS.

### GENERAL HOSPITALS—*Continued.*

are being made to improve the accommodation for those who are obliged to resort to this hospital in times of sickness and accident. Under the able leadership of the Hon. Sydney Holland, as Chairman, the Committee are striving to make this hospital fully equal to the demands made upon it. They devote their time and energies, but they sorely need the help of the public as contributors to the funds. We strongly urge our readers to help this great charity of the East End. Full information about the institution may be had from the House Governor and Secretary, Mr. G. Q. Roberts, M.A.

**Metropolitan Hospital**, Kingsland Road, N.E.—The need for this hospital in the poor and densely-populated districts in the midst of which it is situated is shown by the fact that both the in- and out-patients have been steadily on the increase for several years past, the numbers treated last year being: in-patients 867 and out-patients 31,245, the attendances of the latter numbering 101,597. There is a debt at the present time of over £3,500, incurred during the past four years. The charity is much crippled by lack of funds, and several beds have had to be closed, leaving only 76 now available for in-patients, although there is accommodation for 160. Secretary, Mr. C. H. Byers.

**Middlesex Hospital**, Mortimer Street, W.—The in-patients for last year numbered 3,611, and the out-patients 49,817. The income from all sources, including legacies, was £31,983, 11s. 7d., and the expenditure £34,557 18s. 9d.; deficiency on the ordinary income and expenditure, £8,296 4s. 3d. The cancer wards are a distinguishing feature of the hospital, and their extra nursing, costly treatment, and unlimited dietary add largely to the expenses of the hospital. Towards the extension of this department, by removing it from the main body of the hospital, and building a new wing, £9,000 is still needed. Secretary-Superintendent, Mr. F. Clare, Melhado.

### THE ARTISAN'S HOME.

### JANET AND JEM.

It matters not how I came to spend my Christmas there, the point is that I did so spend it, and met with a delicate wish on the part of my hosts to make me feel that my being their lodger was a matter of the greatest pecuniary benefit to themselves, whereas Jem Smith, a clever carpenter, was in receipt of good wages, needed no help, and was somewhat incommoded by my establishment in one of his three small bedrooms.

All the artisan class do not resemble my friends Jem and Janet; no, indeed, I saw some sad instances of selfishness, greed and cruelty, but there is much that is lovable amongst them. They are ready enough, especially the women, to say hard things of one another. Mary Jane being the envied possessor of a smarter hat than Clara Anne, Clara Anne suddenly discerns an unbecoming lightness of conduct in Mary Jane; and for disparagement of neighbours' characters, conveyed sometimes in open discussion over garden walls, or by a slight but damnatory sniff over the tea cups, no society dame can surpass them. But if any real misfortune happens to Mary Jane, in seven cases out of ten Clara Anne will come forward with some little kindness and the atrocious hat is forgotten.

The Smiths had five children, the two eldest being boys of eleven and thirteen. They had two decent-sized bedrooms and a slip for the boys—noisy, healthy fellows, but well brought up. The two little girls of seven and five were turned out of their room for me and put in the slip, whilst 'Enery and Alf were relegated to the kitchen. To Janet's tidy soul this was a trial, I know, but with tactful delicacy she assured me that 'Enery " as ran errands and such like out of school hours " wanted his nights " comfortable," and lately the baby had cried and so she was glad to put him further off. The idea of 'Enery, who ate like a schoolboy and slept like a dormouse, having disturbed slumbers was entertaining, but I appreciated the feeling that dictated such deviation from the truth.

In the mornings I helped Janet get her puddings and cakes ready. This occupied some time, for a baby and two small girls are a hindrance, to say nothing of preparing meals for the husband and two hungry boys. Moreover, there were frequent interruptions caused by neighbours, tidy and untidy, calling for the loan of some article of domestic use, or " a pinch of tea " or " a taste of cheese." Marigold Terrace was a row of five-roomed cottages, a kitchen and parlour below, and three rooms upstairs. From their outsides a fair idea of their inmates might be gained. Our house was spotless, with short muslin blinds to all the windows, and a gelatinous wax-fruit group in the parlour. Next door, Mrs. Jones's slatternly habits betrayed themselves in blinds that never hung straight, and smart long netted curtains that fell in melancholy limp loops, sadly in need of the wash-tub.

The lady of this house was a more consistent borrower, than any—her " man " was just coming round the corner and there wasn't " a mossel of tea " in the place—or she was " that caddled with the wash, and wherever her big iron was, she knew no more than the dead " (probably pawned a few days before)!

On the opposite side lived a new-married couple, young and prosperous, but the man already slipping down the drunkard's hill, perhaps the victim of heredity, for his father drank himself to death, and his mother was a confirmed small tippler. The girl was near her confinement, and Stokes was good to her; but already her face had an anxious look.

Janet kept a money-box on the mantelshelf, and into it farthings found their way all the year round for a little jollification at Christmas, such as snapdragon, crackers nuts, &c., and a few inexpensive toys for Santa Claus to drop about.

On opening this in secrecy at night, it was found to yield three-and-ninepence. I saw Janet looking at this wealth, and then at her husband.

" Well, what is it, mother ? " he inquired.

" Why, Jem, there is Amelia ; it seems downright mean for us to have so much and her to have nothing ! "

" All right, mother, you want to give her something ; that's about the size of it, ain't it ? "

" Why, Jem, how quick you are ! " said the guileful Janet. " I was thinking how I could manage, and Miss thinks so too "—turning to me as she spoke, ungrammatical but impressive—" that poor girl's got nothing but a dirty old ulster to lie about in, and that's pretty nigh threadbare ; and we thought, if you approved, we could cut up that old Paisley of Aunt's, as I'm sure I should look a figure of fun in, and with a shilling or two we could line it warm with a bit of flannel in the body and make

### SWEET CHARITY'S GUIDE TO CHRISTMAS GIVERS.

### GENERAL HOSPITALS—*Continued.*

**North-West London Hospital,** Kentish Town Road, N.W., was founded in 1878, and is the only institution of the kind in the north-west district. It has 53 beds, and last year there were 585 in-patients and 18,473 out-patients. Secretary, Mr. Alfred Craske.

**Poplar Hospital for Accidents,** Blackwall, E.—Situated amongst a teeming population of poor hard-working people in a district which may be called the " workshop " as well as the " port " of London, this hospital is doing an excellent work. The demands on this institution of late years have greatly increased, and consequently further subscriptions and donations are very necessary. Chairman, the Hon. Sydney Holland. Secretary, Lieut.-Colonel Feneran.

**Royal Free Hospital,** Gray's Inn Road, W.C.—Having no endowment, this hospital is entirely dependent for support on the subscriptions of its governors and the voluntary donations and bequests of its friends. It admits into its wards over 2,000 poor sick persons annually, besides administering advice and medicine to more than 25,000 out-patients who resort to it, not only from the crowded courts and alleys in the immediate neighbourhood, but from all parts of London and the suburban districts. As is indicated by its title, admission to the hospital is entirely free; no subscribers' letters are required, and no charge whatever is made to the patients. The relief thus afforded is effected at a cost of about £12,000 per annum, while the reliable income does not exceed one-third of that sum. Secretary, Mr. Conrad W. Thies.

**St. George's Hospital,** Hyde Park Corner, S.W.—This institution contains 351 beds, and during 1899 treated 4,711 in-patients, 14,622 out-patients, 14,640 casualties, and 451 maternity cases. It has been recently remodelled throughout and is one of the most deserving and best her a dressing-gown, as she would not look the scarecrow she do now."

Of course Jem thought as she did, and I admired to see how I was drawn into the affair, though never having heard of it till that moment. This matter arranged, Janet and I worked early and late making the dressing-gown for the crippled neighbour who lived a few doors off. It was finished late Christmas Eve, resplendently tied at the neck with grass-green ribbon of a fearsome hue. Jem was instructed to knock at the door, drop the parcel on the step, and beat a hasty retreat, so that Amelia might have all the pleasure of wondering whence it came. The delights of this Christmas were enhanced by the fact that all the family were united. Last year little Polly had been in hospital with diphtheria, and though her life had been spared, and ere Christmas she had been pronounced out of danger, the agony of apprehension the parents had gone through would never be forgotten. Some people, nay the larger proportion in all classes, are prone to receive benefits without a word or sign of thankfulness, and to consider them quite as their due, and as a recognition both from heaven and earth of their exceptional merit. This was not the case with my kind friends. Jem passed the hospital where little Polly was succoured every day on his way to work, and once a week, as regularly as he took his wages, he dropped a small offering into the box for contributions at the gate. He never failed to give a penny, and on propitious days, when 'Enery and Alf were well provided with stout boots, and the little girls lacked nothing essential, then Jem's offering was enlarged; but little or big it was given with a grateful and willing heart, and a comfortable feeling that should he need hospital help again he would have done his humble best when he could. In spite of the Paisley garment and the hospital thankoffering we had our Santa Claus stockings, our snap-dragon, and a gorgeous dinner. Little Polly, aged five, was much awed by the lurid glare of the burning spirit, calling out, in a horrified whisper, "Miss, ugly! ugly! all green!"

On Christmas Eve the thriftless Mrs. Jones arrived for the loan of "a few currants and a mossel of dripping," but Jem was in possession of the kitchen, and curtly replied that there were no currants nor dripping in the house. The lady retired muttering anathemas on Jem's "closeness;" and yet there was plenty of good in that woman. Two hours later, a sad thing befell the Stokes' household. The man came home, as Mrs. Jones expressed it, "blind drunk," and either knocked his wife down or lurched against her so that she fell; at any rate, her baby was born prematurely; nothing was ready and no one near to help. Then Mrs. Jones came to the rescue, slipshod but useful, kind and tender, whilst her husband, also far too fond of the bottle, rose to the occasion and dragged off the wretched Stokes into the Jones's house, and let him sleep off his drunken bout far from his poor young wife.

Then, too, did Mrs. Brown, hitherto at deadly feud with Mrs. Jones on the subject of some missing clothes pegs, also show her best side. I had been over Christmas morning to see what I could do for the invalid when I met Mrs. Brown, always the pink of neatness, carrying in a tray, who thus greeted me:—

"I am just a taking in a bit of dinner for Mrs. Jones, poor soul, as has been up all night, and not a bit of victuals to eat very like. Ours was nice and hot, and it seems unneighbourly to leave her out. Pudden looks good, don't it?" she continued. Then a bit of the old Adam rising to the surface—

"My man says as mine always eats light; but I heve heerd tell," dropping her voice mysteriously, "as Mrs. Jones' puddens sits dreadful heavy on the stomick!"

It has not been my last visit to Jem and Janet, though I have never spent a second Christmas there; but I often return and am warmly welcomed even by Mrs. Jones, who is still chronically lacking a "mossel" of something.

### The Slums.

It was a pouring wet night, and the rain soaked through Mrs. Sims' shawl, and made cascades in miniature from the brim of her old straw hat, as she walked hurriedly down the squalid street in which her home was situated.

Most of the houses bore the impress of extreme poverty, but here and there the monotony was relieved by a tidy white blind or a clean window pane, which suggested that the family within had aspirations or traditions above those of their neighbours.

Mrs. Sims entered the open door of No. 7, and her sodden boots left their outline on every step, as she mounted

### SWEET CHARITY'S GUIDE TO CHRISTMAS GIVERS.

### GENERAL HOSPITALS—*Concluded.*

administered of hospitals. Secretary and Superintendent, Mr. C. L. Todd.

**St. Mary's Hospital**, Paddington, W.—Very heavy work is thrown upon this institution owing to the large area of scattered poor which it has to assist. It is not so well supported as it should be, in spite of, or rather because of, its many wealthy neighbours. The board of management, therefore, urgently appeal for further support. The special wants of the hospital are: (1) New annual subscriptions; (2) donations for the endowment of beds and cots. The hospital is free, and no urgent case is refused admission. Secretary, Mr. Thomas Ryan.

**Seamen's Hospital Society, "Dreadnought,"** Greenwich, S.E.—Entirely supported by voluntary contributions.—During 1899 25,797 sick and injured sailors were admitted to the various establishments of the Society. The branch hospital at the Royal Victoria and Albert Docks is now enlarged to 50 beds. The London School of Tropical Medicine, which is under the auspices of her Majesty's Government, is established at the branch hospital of the Society. Secretary, Mr. P. Michelli.

**University College Hospital**, Gower Street, W.C.—This hospital was founded in 1833, and during the past 66 years has treated over one million and a half patients. In 1899 alone 2,708 in-patients and 37,562 out-patients were treated, 267 patients were sent to convalescent homes, and nearly 800 surgical and other appliances were supplied to poor patients. The financial condition of the charity is at all times a matter requiring the serious consideration of the committee, as the reliable income from all sources is only £8,000, and the necessary annual expenditure is over £18,000. When the new and larger hospital is completed, it is estimated that to keep the whole building in proper working order about £20,000 per annum will be required from voluntary sources. Secretary, Mr. Newton H. Nixon.

the narrow stairs. Before she reached the second landing a shrill voice greeted her.

"Is that you, mother?"

"Yes, it's yer mother, right enough, Nell; but don't you come a-nigh me. I'm as wet as a brook." She unpinned her dripping shawl as she spoke, and spread it over the top rail of the banisters.

"Did you see father, and how is he?"

"Well, he ain't up to much, Nell, and that's the truth; he seems to be frettin' above a bit 'cos he've bin lying in 'orspital so long."

"Did you speak to the doctor?"

"No, he wasn't in; but Nurse give me a message from 'im. He says that there ain't no manner of reason why father should not be as fit as ever he was, by-'n-by, only he ought to stop where he is for another month."

"Oh, mother!"

The dismay in the eyes of the precocious child was reflected momentarily in those of the mother. But the latter soon spoke again.

"Yes, Nell, that's what the doctor says, and so I suppose we must just make the best of it."

"Was father very cross at having to stop such a while?"

"Well, he didn't seem, not to say *real* disagreeable about it, only a bit put out. He asked a sight o' questions, too, and I'd a hard job to make him believe as we was gettin' on pretty comfortable without 'im."

"He'll find out fast enough when he sees as you 've give up the ground floor and took the attic," argued Nell.

"Well, there's time enough for that, ain't there? T'aint a mite o' good meeting trouble half-way, as the sayin' is." And pushing Nell aside, Mrs. Sims proceeded to kindle a fire and fill the kettle.

"Where's the boys?"

"They've both fell asleep on the bed. I'd a job with young Steve: he was cryin' all the time, said he was hungry. I give him the last of the bread, an' then he dropped off. Sammie went right enough when it got dark, and I put me jacket over him. I had to promise as I'd call him when you come back."

Nell started up to redeem her promise to her little brother, but her mother caught hold of her.

"Don't you be a little fool, Nell; for God's sake let 'em sleep. You knows yerself as they're bound to wake up hungry. Just you wait till I've made a drop of tea Sleep's a fine stay for an empty stomach!"

The room was fairly clean and orderly, but the stock of fuel in the dark cupboard would not have filled an ordinary coal-box, and Mrs. Sims stoked her small fire prudently.

"They was just giving father 'is tea when I got there, and it looked proper, I tell ye. There was a bit of a white cloth on a tray, and the tea was streamin' hot, and an egg boiled just so. He looked very hard at me, an' I knew as he was a wishing as I could be served the same. So I up and makes a joke about it, and spoke ever so saucy, askin' 'im to guess what time you an' me had ours to-day."

"Oh, mother, how could you laugh about it?"

"Well, we had a cup o' tea to our dinner, an' there was no need to tell 'im as we'd nothing else."

"Did father tell you what he'd had for dinner?" asked little Nell wistfully.

"Yes; he said they give 'im a slice of mutton cut from the leg, an' gravy an' 'taters and rice puddin' a'ter."

Nell's pale cheeks flushed as she spoke.

"Oh, mother, I feel as I can smell that mutton, can't you?"

"Best not think about it, child. Where's my basket got to? I've brought in a nice crusty loaf an' a bit o' cheese as smells real tasty. Now put a pinch o' tea in the small pot and I'll have they boys awake in a jiffey."

"Father's wanting to see all on you," she remarked as she cut thick slices from the crusty loaf, "so I asked the Sister if we'd be let in Christmas Day. She spoke very kind, she did, and said as I was to bring you along at four o'clock to have tea with 'im. They're a-dressin' up the ward a treat, I can tell you."

"I'd most forgot about Christmas," said little Nell soberly. "Ain't we goin' to have no dinner at home?"

"Where's a Christmas dinner to come from, Nell? Yer poor father's so downhearted I dursn't tell 'im how we're situated. You knows well enough that I loses 'arf a day's work every time I goes to the 'orspital—it's such a way off. An' if I was to miss one of the visitin' days he'd be in a fine way, I reckon."

"I suppose you'll make a long day to-morrow?"

"I'll have to, and most as I earns 'ull go for the rent. Eh, but it's cruel hard on a woman when she's got a sick husband at Christmas."

. . . . . . . . . . . . . . .

At No. 3 in the same poor street there was a short white blind in the ground floor window, also narrow

### SWEET CHARITY'S GUIDE TO CHRISTMAS GIVERS.

### PROVINCIAL HOSPITALS.

**The Birmingham General Hospital.** (Founded 1766).—The new building of this hospital was opened by H.R.H. Princess Christian, on behalf of H.M. the Queen, on July 7th, 1897, and contains 346 beds. To meet the increased accommodation provided a larger income is necessary, and an earnest appeal is made for new and increased annual subscriptions or donations. House Governor, Mr. Howard J. Collins.

### CONSUMPTION HOSPITALS.

**Bournemouth National Sanatorium for Consumption and Diseases of the Chest.**—Patients in humble circumstances who are convalescent, who require further medical treatment and change of air, or who are suffering from an incipient form of the disease, are cases for the relief of which this institution was founded. The "open-air" treatment is carried out completely in all suitable cases. There is at present only accommodation for 31 men and 31 women, but to meet demands, building extensions are being provided to add 10 beds, and for this purpose £1,000 is still required. The committee earnestly solicit liberal support, as this national sanatorium is entirely dependent on voluntary contributions. Secretary, Mr. G. Lowe Riddett.

**Brompton Hospital for Consumption and Diseases of the Chest,** S.W.—This hospital is well known as one where patients are well cared for, and every effort is made to brighten their clouded lives. The committee appeal for new subscribers to replace those removed by death, and other causes, and for assistance to enable them to erect a much-needed "country branch and convalescent home," the site for which has been already secured at Heatherside, near Bagshot, and gifts are also asked for towards the annual Christmas tree. Secretary, Mr. William H. Theobald.

moreen curtains which were drawn, on ordinary occasions, as soon as the evil-smelling paraffin lamp was lit.

But on Christmas Eve Mrs. Higgins was too busy to think of the curtains, for she was dressing up her small room.

Her husband was a labourer, and he sat in clay-stained garments smoking a short black pipe.

"I'm on for a spree, lass," he said, jingling his wages in his trouser pockets. "You get yer hat on, an' let's be goin'."

"I'd a mind to finish this here job." The woman spoke with hesitation, half fearing a rough answer; but when her husband replied he did it quietly.

"I don't see what's the good of it, but please yerself! Say, Sal, it don't hardly seem a twelvemonth since we buried the little 'uns."

The woman began to cry.

"I've been fancying as I could hear 'em call 'Mother,' as they used to. I thought as I should go out o' my mind all day, a-wanting of 'em back. An' then I thought o' something as cheered me above a bit. On'y I'm feared to ask you."

Report said that Mrs. Higgins had cause to fear her husband, for the "sprees" in which he periodically indulged left him morose and irritable. But his wife never discussed his moods with her neighbours, answering when questioned that he "was all she'd got left to care for."

"Now you stop frettin', Sal; I never could abide to see a woman cry. Wipe your eyes, lass, and give us a kiss. The kids is better off, if the parson speaks true."

Encouraged by his tone Mrs. Higgins divulged her plan, but it was not well received at first. The man saw that he would have to relinquish the evening's spree, and also would be called upon to hand over most of the week's money for the entertainment which his wife was set upon giving. His earnings were but small, and he clung to them.

The woman persisted, gaining more courage as she pleaded, and the man yielded quite suddenly at last.

"Have yer own way, Sal; I won't go agin it. You ain't one as asks a favour often." Then, feeling somewhat elated by his own unusual graciousness, he laid the money in his wife's hand.

The day was passing slowly with Nell and her brothers. Their mother had gone out very early. When it grew dusk in the attic the children wandered into the street, where Mrs. Higgins' uncurtained window presently attracted their attention. By flattening their little cold noses against the glass they were able to get a good view of the interior. Festoons of gay-coloured paper were slung across the ceiling, and on the chimney-piece two or three sprigs of real red-berried holly showed up bravely. The children stood entranced till a hoarse voice behind them caused them to start like guilty things.

"What be you little kids a-doin' at my window?" asked Higgins, who had just returned from an errand for his wife.

"We ain't doin' no harm," retorted Nell sharply; "we was only lookin' at all them pretty things. Ain't the room beautiful?"

"Pretty middlin'. Where do you kids hang out?"

"Mother have took the attic at No. 7; father's been in orspital nigh on three months."

"Blest if you ain't poor Sims' kids as my missis was jawing about. Well, that's a rum thing too. Just you come in and see what she'll say to you."

But the children hung back.

"Oh, please do let us run away! We ain't done nothing. We'll go straight home," pleaded Nell, clutching a boy with either hand.

At this moment Mrs. Higgins appeared in the doorway.

"Yes, them's the kids, but don't you tell them nothing to-night. I'm goin' to see their mother."

. . . . . . . . . . . . .

Mrs. Sims was mending the children's clothes late that same evening when a knock was followed by the entrance of Mrs. Higgins. She wore a shawl over her head and was a little breathless from hurrying up the stairs.

"I'm a bit late, I know, but you'll excuse me, won't you? I've had a many things to see to. I've been doin' a bit o' shoppin', to tell the truth. I've just dropped in to say as me and my husband 'ull take it very neighbourly if you and your young 'uns will eat your Christmas dinner along of him and me. There won't be nothing grand," she added anxiously "but there's a piece o' beef as looks as tender as you'd wish to see, and greens and 'taters and a pudding."

"I'm sure you're very kind to think about it, but there, I really couldn't do it. Thank you all the same. We ain't so short o' things as you might fancy," she added, with a piteous attempt to assert her independence, and

### SWEET CHARITY'S GUIDE TO CHRISTMAS GIVERS.

### CONSUMPTION HOSPITALS—*Continued.*

**Cancer Hospital.** Free. Fulham Road, S.W.—Founded in 1851 for the free treatment of the necessitous poor who are afflicted with cancer, tumours, or allied diseases. The nature of the disease makes it necessary to supply both food and dressings in greater quantity and higher quality than is necessary at a general hospital. The annual expenditure amounts to about £12,000, to meet which there are annual subscriptions of £2,000 and dividends £3,400. The balance has to be made up by appealing for donations, &c., and generally it means selling out stock to the amount of about £3,000 or £4,000.

**North London Hospital for Consumption and Diseases of the Chest,** Mount Vernon, Hampstead, N.W., and Fitzroy Square, W.—The "open-air" treatment of consumption is carried out at this institution, which is admirably situated on the hills of Hampstead, some 400 feet above sea level. The results of the adoption of this method of treatment are so far most encouraging, and the committee have erected special balconies in order that the treatment may be extended. There are at present 80 beds in the hospital, and the committee appeal for funds to enable them to add another 20 beds at the commencement of the coming year. Secretary, Mr. William J. Morton.

**Royal National Hospital for Consumption and Diseases of the Chest,** Ventnor, Isle of Wight.—A new block for 21 additional men patients was opened in 1899, and the hospital now accommodates 153 patients. Every bed is occupied, and 209 approved candidates are waiting their turns for admission. As the expenses exceed the assured income by £4,000, the committee appeal for additional annual subscriptions and donations. Secretary, Mr. Ernest Morgan. London office, 34 Craven Street, Charing Cross, S.W.

**Royal Hospital for Diseases of the Chest,** City Road, E.C.—The expenditure at this hospital exceeds £8,000, an effort to hide the tears which welled up in her poor tired eyes.

"Oh, Lor'! whatever do it matter? We've all had trouble. Look at me. Last Christmas I'd my children with me, same as you; and in the New Year they was taken from me, both in one week. They took the diphtheria so cruel bad the doctor couldn't save 'em. You're better off than I be, ain't you? I've only got my husband left me."

"I'd like to come and cheer you up a bit to-morrow, but with the three children it makes such a party to trespass on you, and we're strangers to your husband."

"Well, if you don't come t'will get me into trouble with 'm, and so I tells you, straight! He give up his Christmas spree soon as I told 'im that t'would be a cruel shame to go and drink a whole week's wages, and let you and your kids go without a dinner. Higgins behaved real 'andsome soon as I made 'im see the sense of it."

It would be hard to say whether hosts or guests looked most contented when they sat down, under the paper garlands in No. 3 on Christmas Day.

Poor Sims lost for a time his anxious expression as his wife related to him, over tea in the ward, full particulars of the hospitality extended to her and the youngsters by their childless neighbours.

As Mrs. Sims left the hospital she dropped two of her hard-earned pence into the box, muttering softly, "Perhaps they'll go to help some as is poorer than me."

### Christmas in the Hospital.

### DIAGNOSED BY THE NURSE.

THERE is a small microbe which constantly infests the wards of all adult hospitals. It is not even mentioned in the text books, and has never been found to flourish in a children's ward. So far it has received no scientific baptism or recognition, but every skilled nurse readily diagnoses the resulting disease even in its most obscure forms.

"Home worry" is the lay name for the painful trouble, and it undoubtedly forms a very serious complication and drawback in many illnesses.

During Christmas week it commonly assumes an epidemic type, and all the cheerfulness of all the ward nurses is needed to banish the bacillus of "fret" from the minds of mothers with a young family left at home in a "two pair back."

"What, another bad night, Mrs. '6'?" asks the bright and sympathetic staff. "Remember there will be no Christmas cake or presents for bad sleepers!"

"You see, it's like this, nurse," says Mrs. "6," apologising for her shortcomings as to sleep, "I can't help hearing the baby cry in the night!"

It wasn't a ward baby—that would have been comparatively easy to cure. It was her own little one down in a Whitechapel court miles away from the hospital. And this is the onset of an attack of "home worry."

Recognising the serious handicap to sick women which lies in the "mother's microbe," I have often speculated on the fine field offered by a large hospital for a new type of workers.

There are so many women in this big city of ours who have little or nothing to do, who find time hang heavily on their hands for want of some good, wholesome occupation.

There is plenty of room for a band of Home Missioners to act as envoys between the hospital mother and the family she has left behind her in crowded court or slum. At this season there are always many willing volunteer hands ready to decorate the ward Christmas tree or to help sister in the voluminous packing of presents for her patients.

There are plenty of willing recruits to perform at the ward concerts, and an army of cheerful helpers to hand round the cakes at the delightful Christmas teas, which afford such pleasant memories to generations of hospital patients. Few or none offer to play the part of "good fairy" or feminine Santa Claus to visit the homes of anxious ward mothers take a trifle of Christmas cheer to neglected little children, and bring back a bit of good news to the patient with an attack of "home worry."

### SWEET CHARITY'S GUIDE TO CHRISTMAS GIVERS.

### CONSUMPTION HOSPITALS—*Concluded.*

towards which there is an annual subscription list of some £2,250, and dividends amounting to £120. £10,000 is urgently needed to acquire a site, build and furnish a nurses' home, and to carry on the work of the hospital. Donations will be gratefully acknowledged by the Secretary, Mr. John Harrold.

### LYING-IN HOSPITALS.

**Queen Charlotte's Lying-in Hospital,** Marylebone Road, N.W.—This hospital received 1,150 patients into its wards last year, and, in addition, attended 1,011 patients at their own homes. The enlargement and renovation of the hospital is now almost completed, and much-needed additional accommodation is now available. The new nurses' home, which has been erected opposite the hospital, and was opened by T.R.H. the Duke and Duchess of York last year, is now in occupation. Upwards of £7,000 is still needed for these works and for new furniture for the hospital and home. Donations to the building fund, as well as for general maintenance, will be thankfully received. Secretary, Mr. Arthur Watts.

**City of London Lying-in Hospital,** City Road, E.C.—Funds are urgently needed to carry on the general work of the hospital, and also to defray the cost of building new dormitories, which the committee have now provided for the nurses and pupils at an outlay of over £4,000. Secretary Mr. R. A. Owthwaite.

### HOSPITALS FOR EPILEPSY AND PARALYSIS.

**National Hospital for the Paralysed and Epileptic** (Albany Memorial), Queen Square, W.C.—The total number of beds provided at this hospital and its Finchley branch is 200, but the accommodation is still painfully insufficient to meet the requirements, as the large majority of patients are

"Mrs. 6" was suffering from the bacillus of baby anxiety. Her neighbour has her husband "on her mind." "He's a good man, Nurse, when he keeps away from the drink; but at Christmas and holidays there's a many temptations and pals 'treating' more than is good for him. And who's to look after the children if he goes on the drink?"

But presently "her man" comes along this Christmas day—quite sober—and the baby turns up crowing and gurgling from Whitechapel, and the row of sick mothers hearten up and forget their pains and weary worryings.

After all, Christmas in hospital is perfectly delightful, with presents and kindliness and bright carols, and everybody forgetting to keep count of the visitors. And Nurse has conjured up from somewhere a bit of Christmas cake and a packet of sweets for the children. For this is a hospital where generous latitude is allowed in the matter of visitors on Christmas Day. It's a poor Christmas keeping for anxious patients when wives, husbands, and little ones are sternly shut out by the closing of the big hospital gates on this great day of the year.

"Please, sir, mother's got to spend Christmas in the hospital, and I've come to see her," timidly said a little girl at the entrance gates of one of our leading London hospitals.

"No visitors admitted on Christmas Day," answered the big porter kindly enough. "It's the strictest rule of the hospital."

And the very poorest rule a hospital ever made, thought the writer, who saw dozens of visitors sent comfortless away on "Christmas Day in the morning" from the unrelenting gate.

But there is a husband and father's microbe, too, which takes the form of worry about ways and means and groceries, and whether the landlord will wait so many weeks for his rent. Here is a poor father of a family laid by with rheumatic fever. He had not been provident enough to join a sick club. His term in hospital is therefore a wageless season—those long, long weeks of helplessness and pain. He meant to do the best for his wife and little ones, but he was careless—he never thought of sickness or to-morrow.

And now he lies tossing about in the long hours of the quiet night, when the still small voice speaks to the most careless and callous. He thinks of the Christmas beef clubs, of blanket and clothing clubs, for which he might have spared a trifle. What a different Christmas it would have been to the wife and children: to the wife who "rough chars," while her man is laid up, and to the children who fend for themselves, and go cold and hungry many a time while mother is "out to work."

But here is nurse, who never forgets the feed time of a sleepless, restless man. And that mug of hot milk makes things look brighter and—— "What, nurse? The doctor thinks I'll soon be going out? And I'm to be sent to a Convalescent Home—and new clothes from the Samaritan Fund? And what an ungrateful beast I was to grumble about the home when it's all coming right, and I'm nearly well!"

"A hospital seems to bring out the small bit of best in a man," said a 'longshoreman—not a very reputable character in everyday life—to the writer. "It's the quietness that makes a cove put his thinking cap on."

Rich people who can pay for the luxury of quiet—and there is no greater luxury that money can buy—regard a hospital ward as a "very noisy place to be ill in."

To the poor of crowded London, whose lives are lived in the publicity, the noise and friction of overcrowded homes, in the strife of the streets and the busy workshop, the hospital ward proves a haven of peace and quietness.

This restful atmosphere sets many a man and woman to think. And the attack of "home worry," so hard to bear at the time, in addition to the pain and weariness of a sick-bed, often results in a sturdy moral convalescence, and a new ideal of home and family, that might never have been reached save through the experience of a "spell in hospital."

It often happens that a new tenderness to wife and children dates from a bad accident or a serious illness. The constant toil and physical activities of a working man's life leave little leisure for reflection; a tenement mother has no time in her daily round for the higher luxury of "thinking things out."

So that the microbe of "home worry" is often a big blessing in disguise. And the tender, ready sympathy of a nurse who recognises the symptoms of this unclassified disease, and bears with it and helps its victim through the dark hours and cheers him over the stony ways that lead to the higher places, is a bigger blessing still. Let no nurse be so scientific and "professional" as to overlook the symptoms of "home sickness" and "home worry."

### SWEET CHARITY'S GUIDE TO CHRISTMAS GIVERS.

### HOSPITALS FOR EPILEPSY AND PARALYSIS

*Concluded.*

unsuited to general hospitals. Many urgent cases are always waiting for beds. Besides the hospital for treatment, there is a pension fund for the incurables. The annual expenditure is £17,000, of which upwards of £10,000 must be raised in benefactions. Director, Mr. B. Burford Rawlings.

### CHILDREN'S HOSPITALS.

**East London Hospital for Children and Dispensary for Women,** Shadwell, E.—It will be readily understood that the struggle for existence in such a neighbourhood as Shadwell is very great, and for this reason the committee of the East London Hospital for Children appeal to the residents in wealthier districts for funds to enable them to help those who are unable to help themselves. The seaside branch at Bognor (28 beds) for convalescent patients materially increases the usefulness of the charity, and at the same time its need of support. Secretary, Mr. Thomas Hayes.

**North-Eastern Hospital for Children,** Hackney Road, Bethnal Green.—Founded 1867. A great effort is now being made to obtain the funds for the comprehensive scheme of enlargement which the committee of this institution have been compelled by force of circumstances to undertake. £8,000 has been spent in purchasing the freehold of two essential building sites adjoining the hospital, and the buildings to be erected upon them are estimated to cost £40,000. The committee, having £5,500 left after spending the £8,000 above mentioned, have recently issued an appeal for £34,500, the balance required to carry out their scheme. The committee propose to build in the first place some extensive premises facing Hackney Road, containing wards for 56 additional beds, a new operation room and new accident rooms, besides other much-needed accommodation. This building is expected to cost £25,000 complete. Further

### THE LABOURERS AT WORK.

CHRISTMAS in a hospital has to many a dreary and incongruous sound, suggesting in painful contrast the extremes of mirth and sadness. For of all the festivals of the Christian year, Christmas holds the first place in our country in point of social observance. It is deeply wrought into our national life and character. The present generation may be more cynical and blasé than its forefathers, nevertheless we keep up the fiction, if not the reality, of "A Merry Christmas." But the hospital is the abode of pain and sickness, and over it hovers the Shadow of the Angel of Death. Does it not seem that the ancient benediction must be a mockery here?

Yet "things are not as they seem." I believe that nowhere is Christmas "kept" with heartier goodwill, more looked forward to, and more thoroughly enjoyed than in the wards of our hospitals. The inevitable monotony of sickness makes the break in the usual routine more welcome, and the spirit of gaiety once aroused is infectious. There must always be some who are too ill to take part in the general festivity, but all who have made any advance towards convalescence are brightened and cheered thereby. Were any churlish Scrooge-like individual to visit one of our large hospitals on Christmas Eve, we might have a modern version of the Christmas Carol.

Nevertheless, the season would be a dreary one to the patients in our hospital wards, but for the efforts of those whose duty it is to minister to them. We all know that pain and sorrow are harder to bear in times of common rejoicing. The separation from home and friends, the anxiety and longing of the mother for her children, of the wife for her husband are more keenly felt at this season. Therefore, those who have charge of the sick, feel it incumbent upon them to do all that is possible to make Christmas bright and happy under the adverse circumstances. Surely this is an aim which, judged by its results, is not unworthy of being classed among the highest efforts of the healing art.

Upon the resident staff, nurses and doctors, and students (if any) lies the responsibility. (I apologise for giving the second place to the doctors who, in point of professional precedence, should come first, but in this case I think the nurses are entitled to first honourable mention.) The hospital funds will not provide for superfluities. Usually the managers have to make urgent appeals for funds to carry on the absolutely necessary part of their work, therefore we depend for our Christmas decorations and entertainments upon the nursing and medical staff. The cost may be met in part by gifts in money and kind from outside friends, but all the work of organisation and invention, and the actual manual labour involved, rests with the *interne*.

And the last item is considerable. The regular work is heavy enough, and is by no means lightened about this time. The fog and ice of December result in many accidents which make the surgical wards heavier than usual, and the same causes operate in producing a plentiful crop of chest diseases which go to swell the total of cases in the medical wards. Many a hospital sister finding her wards full, with perhaps three or four very critical cases demanding closest attention, may well feel her heart sink with dismay at the thought of the extra work which Christmas brings. But such apprehension is rarely shown and is generally bravely put aside.

Under any circumstances the task, in addition to the ordinary duty, of planning and executing an elaborate scheme of decoration and arranging for the concert, tableaux, waxwork exhibition or dramatic entertainment, throws a serious strain upon the workers. Visitors who are admitted to the wards at Christmas say admiringly: "How pretty! Really the effect is charming! Who has done it all?" And when told that each little company of nurses has been responsible for the decoration of their own ward, the reply perhaps is: "Indeed! It must have taken a great deal of time." But few have any adequate conception of how much time has gone to the making of those wreaths, devices, and mottoes, nor of the sacrifice, albeit a willing one, of personal leisure and even the expense in money which is entailed.

The nurses begin their preparations for Christmas several weeks beforehand, arranging their plans and working at the less perishable part of the decorations. For it must be remembered that all has to be done in odd moments or when off duty. Obviously, the regular work cannot be slurred or neglected, therefore some nurses give up their precious two hours "off duty" nearly every day for two or three weeks before Christmas; but such over-zeal, even in a good cause, is not to be commended. It is well to begin early, but yet there is of course much that cannot be done until a few days before Christmas. This is the time of

### SWEET CHARITY'S GUIDE TO CHRISTMAS GIVERS.

### CHILDREN'S HOSPITALS—*Concluded.*

help is urgently needed. Secretary, Mr. F. Glenton-Kerr.

**Paddington Green Children's Hospital.**—This hospital was established in 1883. It was rebuilt and enlarged in 1895. It provides 46 cots. There is a large out-patient department, where over 1,000 patients are treated every week. The hospital has a Convalescent Home for twelve children at Wealdstone, Harrow. Funds are urgently needed for the maintenance of both the hospital and the home. The hospital owes its bankers £1,500. Secretary, Mr. W. H. Pearce.

**The Hospital for Sick Children,** Great Ormond Street, London, W.C.—The Hospital for Sick Children is the oldest and largest children's hospital in the British Empire. £20 a day is required to meet the difference between expenditure and income. Over 2,000 in-patients are treated annually. New subscriptions are really required. Debt, £24,000. Secretary, Mr. Adrian Hope.

**Victoria Hospital for Children,** Queen's Road, Chelsea, S.W. (and Victoria Convalescent Home, Broadstairs).—The committee are appealing for special donations to the fund for the erection of a new building at a cost of £25,000. The hospital further seeks subscriptions to help it to carry on its work. Secretary, Mr. H. G. Evered.

### HOSPITALS FOR WOMEN.

**Chelsea Hospital for Women,** Fulham Road, S.W.—This hospital (52 beds) is entirely without endowment or reserve funds, and greatly needs legacies and new annual subscriptions. There is a Convalescent Home (22 beds) at St. Leonards-on-Sea, but it is not restricted to those who have been patients in the hospital. Secretary, Mr. Herbert H. Jennings.

high pressure which has its climax on Christmas Eve. All available help is now eagerly sought for. Up to this point usually all the work has been done by the nurses with such help as they could obtain from convalescent patients. But when it comes to the actual "putting-up" of the decorations, we find the advantage of able-bodied masculine assistance. The help of the students is invaluable at this stage, and even a good-natured house surgeon often does not think it beneath his dignity to take off his coat and "lend a hand."

In the subsequent entertainments the medical staff take a full, often the principal share. Again, how much skill and patience, how much organising and rehearsing is necessary! But the results, which often rise above the level of ordinary amateur efforts, fully repay as a rule by their success and the pleasure given, the trouble they cost. Too much may be attempted at such a time. If the strain beforehand has been so severe that the workers are tired out ere Christmas comes, or if the expense has been such as to be tax upon them which they can ill afford, then it were far better to revert to simpler modes. There is much to be said for the view of the chairman of one of the largest London hospitals who has been instrumental in banishing decorations from his wards in the interests of the nurses. But yet we women love pretty things and generally cling to the good old custom of Christmas decorations. It is not necessary to give them up if we study to observe moderation in this as in all things.

### THE TRANSFORMATION.

The grand transformation scene has been effected. The tired workers now "rest from their labours" and survey with pride the result of their handiwork. The last holly and ivy leaf, piece of string, paper, litter of all kinds has been swept up, and the nurses prepare to go to bed, knowing full well that they will be aroused early by the strains of the carol singers.

"Christians, awake! Salute the happy morn."

"Oh dear!" was the irreverent comment of one weary nurse, "I wish they'd let a Christian sleep!" Yet, in our hearts we all cherish the ancient customs, and love to hear the old carols "so early in the morning."

On Christmas Day the ordinary rules and restrictions are relaxed, and the nurses in the intervals of duty visit each other's wards, admiring, criticising, comparing. Even some of the patients who are sufficiently convalescent join in the tour of inspection. Let us follow in imagination in their train.

The entrance hall claims first notice. It is beautifully decorated with festoons of evergreens, plants, and miniature shrubs, and hung with flags and Chinese lanterns. Over the central archway is displayed in frosted letters the greeting "Welcome to all." To the right and left are the corridors leading to the wards. And how lovely the latter appear in their festal array! It is difficult indeed to judge between their rival attractions. In one hospital where I was staff nurse the chairman, a wealthy and generous man, to whose liberality the hospital owed much, once offered a prize for the best decorated ward. Of course the competition was keen for the honour, and the nurses surpassed themselves in the results of their efforts. When all was finished the hardest task remained, viz., to decide between the rival wards. Our chairman professed himself quite unable to do so, and probably felt it invidious to make distinction where all the workers had done their best, and each scheme included some special points of excellence. The final judgment was eminently satisfactory. Three wards were placed in the first class, but *all* were highly commended, and each received a prize!

Continuing our tour of inspection we find that while there is a general air of uniformity, each ward has characteristic distinctions. One, for instance, has a specially-gorgeous collection of Chinese lanterns and Japanese curios. Another makes a brave display of bunting. Many of the flags own no nationality whatever, but we are not critical and pass on. A third is brilliant with many colours, having utilised coloured paper largely in its decoration. Paper flowers—roses, camelias, chrysanthemums—are interspersed among the greenery, and wonderfully ingenious and life-like at a distance those flowers are! They are the work of one of the patients, a pale anæmic girl who is an artificial flower maker by trade. There are also paper chains, made of innumerable links of many colours, festooned from point to point in conjunction with wreaths of evergreens. Then we come to a ward the sister of which has declared that, owing to bad cases, and a consequently

### SWEET CHARITY'S GUIDE TO CHRISTMAS GIVERS.

### HOSPITALS FOR WOMEN—*Concluded.*

**Samaritan Free Hospital for Women and Children,** Marylebone Road, N.W.—Some £6,000 per annum is required to maintain this hospital, of which only £1,600 can be relied upon in annual subscriptions. The committee would, therefore, like to see a great addition to the list of annual and life subscriptions. Secretary, Mr. W. G. King.

**The Hospital for Women,** Soho Square, W.—There are 61 beds in constant use, and, as the hospital possesses no endowment, funds for their maintenance are much needed. The committee very earnestly appeal for additional annual subscriptions. Secretary, Mr. David Cannon.

### MISCELLANEOUS—SPECIAL.

**Dental Hospital of London,** Leicester Square, W.C.—The present hospital, which has been enlarged to its utmost extent, is quite inadequate, both in accommodation and sanitation, for the large number of patients. A new hospital is, therefore, being built on a very suitable site, almost adjoining the present hospital, which, it is hoped, will be completed by the spring of next year; and funds, for which an appeal has for some time been before the public, are still urgently needed. Secretary, Mr. J. Francis Pink.

**London Fever Hospital,** Liverpool Road, Islington, N.—The institution is dependent upon voluntary support, and donations and subscriptions will be gratefully received, especially as the alterations and additions to the hospital now being carried out, and the building of a convalescent home, are taxing to the utmost the resources of the institution. Secretary, Major W. Christie.

**London Lock Hospital and Rescue Home,** Harrow Road, W.—Help is required for this valuable institution. The present year has not been a good one financially. There has been a continual flow of patients to the hospital, some heavy pressure of work, she cannot undertake elaborate decorations; yet this ward is not less charming in its simplicity than the others. There is little wreathing, or decoration of any kind on the walls, and no mottoes but the one customary Christmas greeting. But the tables have bright-coloured "centres," on which are grouped plants in art vases, and there are bouquets here and elsewhere about the ward of holly, frosted ivy (easily done), and evergreen branches. Also a few flowers in slender glasses, scarlet geraniums and pale narcissi. The money which would have been spent on decorative materials has been spent here on additional plants for the ward, which will be things of beauty and a joy, if not for ever, yet long after the other decorations have departed. Contrasting the small amount of labour expended here with that given in other wards for a transitory result, we are inclined to think that, considering all things, this sister has chosen the wiser part. The children's ward is always the most attractive in a hospital, and is so now. Cots are more picturesque than beds, especially when draped with white curtains, which are tied back on this occasion with bows of bright-coloured sash ribbon. The tiny occupants look so charming, too, in scarlet jackets if in bed, or if up, resplendent in smart pinafores, and gay sashes, "tie-ups" and hair ribbons, saved for Christmas! The principal feature of the decorations here is the magnificent Christmas tree, reaching to the ceiling and laden with toys and glittering spangles. To-morrow the toys will be distributed; for to-day the tree remains undespoiled of its wealth, a source of wonder and joyous speculation to all the children. The motto over the door of this ward is from Whittier—

> A dreary place would be this earth
> Were there no little people in it,
> The song of life would lose its mirth
> Were there no children to begin it.

And facing the entrance is a scroll bearing for inscription a verse of the children's hymn, beginning,

> There came a little Child to earth
> Long ago.

The subject of mottoes affords scope for originality every year. Of course, there are the old-fashioned and "seasonable" ones, but we like others, new and suggestive. Some one of a facetious turn must have been responsible for the inscription in one of the principal medical wards, over the medicine cupboard "It is more blessed to give than to receive!" Lofty, spacious, well-lighted wards make a good groundwork for the decorator's art, but some wards in our old hospitals are small, dark, and irregularly-shaped, and consequently put the skill of the workers to a greater test. One of our illustrations shows a ward in the roof of one of the principal London hospitals, which certainly must have seemed difficult to treat decoratively. Yet by following the converging lines of the roof, a tent or bower-like effect is produced, which is picturesque, and attests in a striking manner the ingenuity of the decorators who have made the best of unpromising conditions.

### Our Wards at Christmas.

We can all predict with tolerable certainty what the average visitor will say on coming round the wards of any hospital at Christmas time. The various exclamations of surprise and delight are agreeably familiar to us. They all have the same keynote, and are all variations of the same tune. We listen; we acquiesce; we are glad to hear their expressions of admiration. We think the ward looks nice ourselves, and our heart swells with a lawful pride. For a brief period our surroundings look like one of the illustrations in a Christmas number, where a highly-decorated hospital ward often forms a suitable background for a short story or Christmas romance. We know what those illustrations are like—festoons and garlands, mottoes and devices, smiling babies in gorgeous cots, fancifully-dressed nurses in uniforms and chatelaines that would be tolerated nowhere—and the imagination of intending probationers is fired thereby with an insatiable longing to become a hospital nurse.

It all looks very charming and dainty to outsiders who only see, as it were, the finished article turned out for public inspection. We, who are behind the scenes and in the hurly-burly, who "press the button" and "do the rest" as well, who have to find for our Christmas decorations money and time, ideas and helpers, to be head and hands at the same time, to strive for order, forethought, and good humour, and exercise in seasons of highest pressure the temper and tact requisite to oil the human machinery and keep it going, without friction, on the one

### SWEET CHARITY'S GUIDE TO CHRISTMAS GIVERS.

### MISCELLANEOUS—SPECIAL—*Continued.*

of which have found their way to the home, in several cases with very good results. It is the desire of all connected with the hospital and home to do all in their power to succour those who are in want of a helping hand.

**Royal London Ophthalmic Hospital,** City Road, E.C.—This hospital, like all hospitals, has been hard hit during the past year owing to the other claims pressed on the charitable public. But its position is different from most other hospitals, because it has not a single penny of invested funds which can be realised. The committee are most reluctant to close a hospital which has been working for nearly one hundred years, which has a world-wide reputation, and which relieves about 40,000 patients annually. The new building in City Road is perfectly designed and equipped for its special object, and worthy of the work and the teaching of the oldest and largest Eye Hospital in Great Britain. On these grounds the committee appeal for help, and beg that contributions may be sent in time to save such a public calamity as the closing of this great institution. Secretary, Mr. Robert J. Bland.

**Royal Orthopædic Hospital,** 297 Oxford Street, W., and 15 Hanover Square.—During the year very great improvements have been made in this hospital. The drainage system has been entirely reconstructed, and extensive alterations carried out in the wards and nurses' quarters. The buildings are now in a thoroughly sanitary condition, besides which, improvements in the wards have added greatly to the comfort of the patients and to the more efficient working of the institution. The committee require the sum of £1,000 before the close of the year to defray outstanding balances in connection with this work, and also to meet tradesmen's bills due to Michaelmas. They ask for a share of the Christmas gifts of the benevolent to enable them to disburse these charges during the current year.

hand, or laxity of discipline on the other—we know, only too well, that Christmas decorations mean anything but unmitigated pleasure. We know that never, all the year round, do we get so tired, that our feet never ache so badly, nor are the hours on duty ever so long as during the Christmas season. And yet, for all that, year after year our enthusiasm rises fresh as ever, and each strives just as mightily for the success of her ward decoration when the time comes to do it, as though last season's fatigue had never been and could not be repeated.

As probationers, perhaps we were not quite so eager about it as in later days. We were not the proud possessors of wards, and we used to get just a little weary of unlimited sweepings away of scattered holly and ivy leaves, running endless errands, and making wreaths in all our spare moments. We did a lot of our staff nurse's work for her just then, while she sat—as we thought—enjoying herself, and helping sister with evergreens and holly and coloured lanterns. We took the temperatures, and made the beds, gave the medicines, and did no end of things that generally we were not allowed to do, besides putting on bonnet and cloak and running out at all sorts of weird hours to buy wire and string and hammers and holly. But if we got little credit for what we did, at least our delinquencies passed unreproved, and we followed for the most part our own sweet wills. Also we were not slow afterwards to appropriate to ourselves the encomiums passed on the ingenuity and originality of our decorations, and bitterly resented any remarks tending to imply that other wards were equal to, if not better, than our own.

When we became staff nurses in our turn, then our sense of responsibility and importance anent decoration began to grow. We were careful to keep the ward door shut at all times during the few days before Christmas, in case nurses of other wards should seize upon our designs, and expand and glorify them into something better. We maintained a dignified silence in the dormitories about our plans, and we were in sister's room a great deal, her faithful aide-de-camp and indefatigable helper in all things.

And, oh, when we first held a sister's post ourselves! How terribly afraid we were lest other wards should outshine our own! What sums we spent on quite needless things, and on what extravagances we launched, for fear we should run short of something the lack of which would spoil the effect of our ward at the critical moment! Some of us who have done this sort of work for many successive years now can look back and smile on the memory of the grand muddle we used to get into every Christmas. With some people, decorations seem synonymous with a glorious mess: you cannot enter their wards without being tripped up by lengths of wire, prodded by unexpected branches of holly in unlooked-for places, and you are never able to find a single legitimate article of ward use among the general chaos. A certain amount of forethought will in a great measure prevent this. So many people do not begin their preparations soon enough, and so from the twentieth to the twenty-fifth of December their wards are a scene of the wildest confusion.

It is not too soon in October to be looking over last year's stock of lanterns, &c., to see what will be wanted for this. Get out some scheme, however rough, of what you intend doing, on paper, and reckon how many lanterns you will require, how many fairy lamps, and how many yards of wreathing will be wanted. Torn lanterns, broken lamps must all be discarded, for shabby decorations are worse than none at all, and new ones bought early, and safely stowed away, not waiting until Christmas Eve, when the probabilities are that the shops will be sold out, or else that you have to pay an exorbitant price for them. Night lights also can be stocked early, to be put in the fairy lamps, and are best bought by the gross. Some people always hire what they want in the way of fairy or standard lamps, candlesticks, etc., but if you do, order them early, and be sure to keep a strict list of all you buy or hire, with their prices. It is a little extra trouble, but will repay you on the next occasion, when you can simply refer to last year's list, to know what will be wanted for this. If you are in a hospital where you are likely to have several men-helpers, then it is well to have plenty of wire beforehand, so that the various lengths can be put up early, and be ready for the wreaths to be attached to them as soon as they are made. Fairly thick wire will be wanted to go from corner to corner of the ceiling, to support the weight of the wreaths and lanterns. Then you will want more or less finer wire, according to the number of the fairy lamps, &c., you are going to put up. If you have a convenient room or cupboard, it is a good plan to get all the wires fixed on your lanterns, and have them ready for use, before you begin making wreaths. Do not forget to have a hammer, plenty of tacks—or needle points if tacks are not allowed—a pair of pincers, and an abundance of string. Old pairs of kid gloves save one's hands, and if

### SWEET CHARITY'S GUIDE TO CHRISTMAS GIVERS.

### MISCELLANEOUS—SPECIAL—*Concluded.*

**Surgical Aid Society,** Salisbury Square, E.C.—The net expenditure for the year amounted to £14,695, of which 84 per cent. was for actual relief. During the past year the large total of 25,219 appliances were given away. There is ample scope for very considerable extension of these benefits, and, therefore, the committee earnestly appeal for contributions. Secretary, Mr. Richard C. Tresidder.

**The Royal Sea Bathing Hospital** (founded at Margate, 1791).—This hospital was founded for the relief of tubercular and other diseases requiring sea air and sea bathing in addition to skilled medical and surgical attention. The system of open-air treatment has been carried out for many years past. The hospital is open all the year round, and receives patients from all parts of the country, but especially from London. £2,000 is urgently needed to pay the accounts due at Christmas. Secretary, Mr. A. Nash, 20 Charing Cross, S.W.

**Western Ophthalmic Hospital,** 155 Marylebone Road, W. At present existing in a private house, quite unfitted for hospital purposes and growing needs, the necessity for erecting new buildings has become urgent, and Sir Reginald Hanson, treasurer to the hospital, has issued an appeal for funds towards this desirable end. Help is earnestly asked for. Secretary, H. A. Dunn.

### GENERAL CHARITIES.

**Association for the Oral Instruction of the Deaf and Dumb.**—The first association to publicly introduce into the United Kingdom the German or pure oral system for teaching deaf and so-called dumb children to speak *viva voce*, and to understand the spoken words of others by lip-reading. The expenses which are very heavy, are met by voluntary contributions and fees, which fall short of what is needed by about £500 per annum. Offices, 11 Fitzroy Square, W.

you have any patients able, you will generally find that they will like helping to make wreaths by sewing holly leaves and small sprays in lengths with large darning needles threaded with fine string. It is light work which can be done in bed. You will be wise, too, if you have a few old pairs of scissors for clipping holly, &c., or you may find the unscrupulous using your best ward scissors for the purpose.

One hospital where I spent Christmas possessed just then an empty ward, shortly to be reopened. This proved a perfect godsend at that time, as into it were brought the various heaps of holly and evergreens, and all the wreaths were done there, instead of making each ward the centre of action. As many nurses as could be spared came during the day to help, and the hospital being as well possessed of large grounds, the wreaths when made were all carried out and laid on the lawn, where they remained until we were ready to put them up in the different wards. They were kept beautifully fresh by this means, and we were able to make them several days before Christmas, so avoiding a very heavy press of work just at the last.

Ward chimney-pieces generally afford ample scope for the exercise of individual talent. This year likenesses of war heroes will be sure to occupy a prominent place, and various representations of "Bobs," with other "Gentlemen in Khaki," will supply centrepieces for many a ward decoration.

But though popular heroes, warlike or political, will be always more or less in favour, the majority of nurses and students still prefer the temporary canonisation of their own pet physicians and surgeons, whose photographs and monograms appear season after season, embellished with many strange devices, and with no end of fairy lamps burning at their shrines. I have seen the initials of one done in fairy lamps themselves, and in another ward the senior surgeon's name was woven into an acrostic by someone of a poetic turn of mind. The verse was printed in letters of white paper on a Turkey red background stretched on a wooden frame, surrounded by greenery, and lamps burnt as footlights on the chimney-piece below it.

One ward possessed a great feature in the form of an enormous spider in an ivy web, and in yet another Chinese lanterns had been excluded altogether, and hundreds of fairy lamps twinkled from the ceiling like stars in the firmament. Where lanterns are used it is just as well not to hang them directly over a bed. Accidents so quickly happen, and even if no great harm is done, the patients are badly frightened by a sudden blaze. Neither should cotton wool be used as ward decorations by those who wish to avoid calling out the fire brigade. It is too terribly inflammable. For lofty wards, festoons of holly and evergreens can be made rather thicker, and hung rather lower between their points of attachment, than those in low-ceilinged rooms; in the latter, thin wreaths of small-leafed ground ivy looks extremely light and pretty, as also wreaths of single holly leaves, threaded on string.

If you are in London, and can possibly get your greenery sent up to you from the country, do so by all means, but if you have to buy it in town, do not wait till the last moment before doing so, or you will have to spend a small fortune on it.

The arms or motto of your hospital also makes a very good centre for decoration, and, of course, the old-fashioned "Merry Xmas" is never out of place. Flags are indispensable, and always effective. They can be arranged in so many ways, and are sure to be more in evidence this year than ever. A scheme of colour as well as form is essential to successful ward decoration, and everything should be made to correspond as far as possible. It is a good plan to have one of the ward tables strike the keynote, and then carry out the colour that is prominent there. Supposing green to be the prevailing tint chosen, then a ward table will look well if covered with a white cloth on which are displayed a few white pots and vases, containing ferns and lilies of the valley with their own delicate green leaves. Trails of smilax, green ribbon and green candleshades will complete the symphony, as it were, and moss can surround the pots and vases.

Red is extremely effective in decoration. Red candle-shades, ribbon, crackers, sweets, with red tulips in white vases, will show up well, also gleaming red holly-berries mixed with their dark green leaves. The lamps and lanterns used, of course, must be red, too, and a table centre of red pongee silk will be needed.

Where there are many student helpers, an archway of stout wire stretched across a ward, covered with ivy, the various points being picked out with coloured lights, has a gorgeous effect, but the work is too laborious for nurses to undertake alone. Cots can always be made into things of beauty very quickly, by temporary curtains of soft butter cloth or art muslin, looped up with bunches of holly and mistletoe, or coloured ribbons, and gold and silver tinsel

### SWEET CHARITY'S GUIDE TO CHRISTMAS GIVERS.

### GENERAL CHARITIES—*Continued.*

**Bethnal Green Free Library,** E.—The poverty in the surrounding neighbourhood compels the committee to make an urgent appeal for funds to meet outstanding liabilities. Contributions will be thankfully received by the Librarian, at the Bethnal Green Free Library, E.; or by the Treasurer, Mr. F. A. Bevan, 54 Lombard Street, E.C.; or Bankers, Barclay and Co., Limited, at same address.

**City of London Truss Society,** 35 Finsbury Square, E.C.—At the present time about 10,000 of both sexes and all ages are treated annually. The premises have recently been enlarged to provide for the increased number of patients, and additional funds are greatly needed to meet the growth in the expenditure. Over 556,000 patients have already been relieved. Secretary, Mr. John Whittington.

**Irish Distressed Ladies Fund,** 17 North Audley Street, W.—Relief is given independently of any question of politics or religion; employment is found, when possible, for those able to work; pecuniary help is given to the aged and infirm; and the education of children is paid for.

**London Orphan Asylum, Watford.**—Owing to an unfortunate diminution in the receipts, a sum of £3,000 is at the present time owing to the Treasurer and the bankers of this institution. This charity is dependent upon public support for £13,600 of the £15,000 required each year for the maintenance of the 500 children in the school. Nevertheless, after anxious consideration it has been decided not to curtail the work of this charity, and 36 children whose cases have, after close inquiry, been found to be really necessitous and deserving of immediate relief, will be elected in January. Subscriptions and donations towards reduction of the debt and providing the means of carrying on the good work in the coming year are earnestly solicited. All particulars will be given by the Secretary, Mr. Henry C. Armiger, at the Office, 21 Great St. Helens, E.C.

causing them to glitter charmingly. Sisters and nurses must introduce a touch of the prevailing ward colour into their uniform, whether as a flower or bit of ribbon, so as to look in keeping, and the patients can wear some, too. Sometimes even a privileged hospital cat follows suit with its collar.

Wards always repay one well for the trouble they give. "When the Christmas lights are all aglow," the last wreath up, the final sweeping done, what sister then, as she stands proudly surveying the result of her care and pains, can possibly regret the time and money she has spent in bringing about such a highly satisfactory result? And if managed well, it can be done without too much being crowded into the last day or two.

Let us, then, keep up the good old custom of decorating our wards. After all, Christmas comes but once a year, and it is our obvious duty to make the season as festive and joyous as we can, to put aside our own heartaches, to keep any sad memories resolutely in the background, while we try to make our patients' Christmas in hospital a bright and pleasant thing to look back upon—an oasis in the desert of sickness and pain—a faint shadowing of the Universal Love that the first Christmas came to breathe upon a sorrowful world.

### CHRISTMAS IN A SCOTTISH HOSPITAL.

THE storm rages outside; swift gusts of wind and snow sweep eddying round the walls of a large hospital in the North of Scotland—typical Christmas weather such as one seldom sees nowadays south of the Border. But within all is bright and cheerful, an expectant air pervades everything. Nurses flit about with trailing wreaths of evergreen and holly. All available help is commandeered by the doctors, and the sombre walls are fast being hidden by bright-coloured mottoes and festoons.

How the eyes of the bairns sparkle and grow large with wonder! No need to tell them it is Christmas Eve. Whispers are heard of "Santie Claus," and one wee boy, well enough to run about and get into everybody's way, whispers to another, "I keekit up the lum [chimney], but I canna speir him yet;" for whatever this very interesting personage may do in other countries, in Scotland he always comes down the chimney. At last all is done, and the day nurses, weary in their labour of love, are replaced by the night nurses, the lights are turned low, the last stocking has been hung up, and sleepy eyes are trying, but in vain, to prop themselves open for the arrival of "Santie Claus."

Christmas morning breaks, bright and frosty; shouts of glee are heard as the bairnies hug their treasures which so mysteriously have found their way into stockings and under pillows; then come the more sober greetings of "A guid Christmas to ye" from the older folk. It is early yet, but further sleep is out of the question. At last seven o'clock strikes, and the day nurses come on duty with kindly smiles and greetings for each one, and, while all work is lightened as far as may be, the ordinary routine of bedmaking and breakfast must be gone through; this over, the doctors and all available nurses gather round the tiny organ on the central landing and the old Christmas favourite is sung, "Hark! the herald angels sing," and a short Christmas service held.

Will the mails be delivered? is the next thought, as anxious glances are cast at the deep-lying snow outside, and a natural one to those who have had experience of the vast snow drifts on the Highland Railway. But at last the letters arrive great excitement is caused by the arrival of letters from the Front, and several are gladdened by news of dear ones who have gone at the call of duty to fight for their Queen and country, and the noble record of Scotland's bravest sons of the "Black Watch" is not likely to be soon forgotten. News also comes from one of the medical staff, and two of the nurses attached to the Red Cross Corps, who have gone it may be to lay down their lives in succouring the wounded and combating with the enteric which has proved so fatal to many of our best and bravest.

But it's an old Scotch proverb "Blue bluid will no' keep awa a funeral if a man doesna gang hame to his denner" and dinner on Christmas Day is always an important item; twelve o'clock comes and roast beef and plum pudding are served out to all who have the good fortune to be allowed on full diet, but there are extra luxuries for all. Then come the visitors, and pain and weariness are forgotten in the greeting of friends and dear ones; the bairns eagerly exhibit their treasures which "Santie Claus" had brought them while they slept, and one old grandfaither is heard to say. "Ay, but there was nae Christmas i' my young days, at least it was'na sae muckle spiered aboot; the bairns

### SWEET CHARITY'S GUIDE TO CHRISTMAS GIVERS.

### GENERAL CHARITIES—*Continued.*

**Mary Wardell Convalescent Home for Scarlet Fever,** Stanmore, Middlesex.—This being the only home for convalescents from scarlet fever, the demand on its 40 beds is great. The committee have been compelled by the District Council to reconstruct the drainage system of the home in order to connect it with the public sewer which has recently been laid down, and funds are urgently wanted to meet this enforced expense. A demand for the second instalment of £200 has been received, but there is no money to meet it. Subscriptions to Miss Mary Wardell.

**North Sea Church Mission,** Quayside, Gorleston, Great Yarmouth.—The operations of this mission, which include religious ministration and medical and surgical relief, are carried on at sea, in the midst of the North Sea fishing fleets. It should be borne in mind that the fishing industry is one of the most hazardous, and it is hoped that the knowledge of the hardship and frequent injury that fishermen encounter may excite the sympathy and support that is so seriously needed by this mission. Chairman, John H. Easterbrook.

**Orphan Working School,** Haverstock Hill, N.W., and Hornsey Rise, N. Offices, 73 Cheapside, E.C.—A national and undenominational institution which maintains 500 children, varying in age from infancy to fourteen or (in special cases) fifteen years. It is greatly in want of funds at the present time. Appeal is made specially for new annual subscriptions, the income of the charity being more than £2,000 below what is needed to meet the expenditure. Secretary, Mr. Alexander Grant.

were'na sae trauchled wi i' my time;" but if you listen you will hear reminiscences of quaint old customs, one being the keeping of "Hogmanay" when visits were paid to all one's friends and neighbours, and at each house Scotch cakes, oaten farls and cheese were brought out, and how all the boys would gather and go from house to house singing a quaint old couplet:

Hogmanay trollolay
Give cakes and cheese and let's away.

And small gifts of money were usually given. It is said that the word "Hogmany" originally meant holy month, and that the custom was symbolical of the visit of the Wise Men, and the offering of gifts to the Infant Saviour.

When the visitors have gone and tea is over comes the great event of the day. The gas is lowered, and one by one the tiny lights flicker on the Christmas tree, which good friends have provided for each ward. The wee bairns are sitting up in their beds with wide open eyes, looking like so many "robin red-breasts" in their scarlet jackets, and there is a general chorus of delight, and, "Ay but its gey bonnie," which if those who had taken so much trouble and thought over the decking of these trees had heard, they would have been amply repaid. But who is this that now makes his appearance? Faither Christmas himself! and willing hands help him to unload the tree, and to each one Father Christmas takes some gift with kindly smile and words, toys for the bairns, tobacco and comforters for the men, shawls, &c., for the women; his voice somehow sounds very familiar to the bairns, and one wee boy, just recovering from a severe illness, and who is a great pet of the house-doctor is heard to remark afterwards, "Faither Chris'mis' eyes were juist like my doctors'," which was very strange wasn't it? Nor are the patients alone remembered, for everyone on the staff of the hospital, nurses, doctors, and all, receive some kindly gift. At last the tree is stripped, and the evening is brought to a close with songs and glees given by the doctors and students.

What a happy day! But Christmas in hospital is never without an element of sadness; in one of the side wards a mother and father watch by the bedside of a dear one, fast bound for the land o' the leal. Their hearts are filled with sadness; but is not the angel's message for them? Surely yes. For only those who watch by the sick and dying realise in its fulness the glorious hope that came into the world—

When the Babe, the world's Redeemer
First revealed his sacred face.

Hark! the refrain of an old Christmas hymn the students are singing reaches their ears, "Gloria in excelsis Deo."

The lights flicker out one by one on the Christmas tree, tired happy bairns sleep with their treasures cuddled up in their pillows, the last "guid nichts" are said, and silence reigns once more. And now, good reader, from Bonnie Scotland comes the message,

A richt guid Christmas to ye all!

### CHRISTMAS IN AN IRISH HOSPITAL.

IT began in the chapel, at seven o'clock—a black, gusty, rainy Christmas morning. We had crossed the road from the Nurses' Home with more than usual care, because of our clean aprons and newly-starched dresses, and we were all conscious of unusual smartness in the way of frilled and laced strings, and of daring deviations from the lines of the plain regulation cap, which we were sure would escape the usual censure—because it was Christmas Day. In our own side aisle we knelt, facing the altar of the Blessed Virgin, with its sweet-faced, blue-robed statue, and its representation of the Manger at Bethlehem, carried out in miniature figures, and lit by many wax-lights.

The high altar blazed with a perfect coruscation of tapers, and glowed with flowers. The nuns of the community occupied the space in front of the altar rails—black-robed, and black-veiled, a hooded band who looked strangely far off and stately to us who knew them in the busy rush of the wards, where they taught us our work with a thoroughness of detail not entirely appreciated by raw probationers, saw to everyone's comfort and considered everyone's feelings (except those of the "pros," who wanted licking into shape!) and held their own well-kept hands from no task, however trying or fatiguing.

Behind the choir of the nuns came the space reserved for the medical and surgical resident staff, who this morning were supplemented by all the visiting staff, resplendent in frock coats and unusually chastened demeanour.

A brilliant choral mass, sung by the nuns, with help from one or two nurses, was performed. Everyone in the

### SWEET CHARITY'S GUIDE TO CHRISTMAS GIVERS.

### GENERAL CHARITIES—*Continued.*

**Royal Albert Orphan Asylum,** Bagshot, Surrey.—This charity has suffered severely in its funds during the past year. The Committee, however, still maintain the full number of inmates. A very novel and interesting scheme is being issued to enable children in better circumstances to aid by small contributions the 200 orphan boys and girls in the Asylum. Full information will be given by the Secretary and Superintendent, Mr. H. W. Tatum, 62 King William Street, E.C,

**Royal Alfred Aged Merchant Seamen's Institution** (established 1867) is the only institution in the United Kingdom which gives, irrespective of rank, ports of service, or place of abode, a home or a pension to the British merchant sailor when old and destitute. Annual subscriptions do not reach £4,000 per annum, and the annual disbursements (including out-pensions and total support of inmates) are £7,000. Secretary, Mr. J. Bailey Walker; office, 58 Fenchurch Street, E.C.

**Royal Association in Aid of the Deaf and Dumb,** Oxford Street, W.—The objects of this society are to visit deaf and dumb persons at their own homes, and to assist them in sickness and distress. During the past year 3,418 visits were paid to the deaf and dumb, 788 visits were made to employers and others on their behalf, 206 were relieved, and 72 provided with work and apprenticed. An increased reliable income is needed to maintain and extend the present work. Secretary, Mr. Thomas Cole.

**Royal Society for the Prevention of Cruelty to Animals,** Founded 1824.—The labour of other charities is divided among many associations, but this charity stands alone—the defender of defenceless dumb animals. In 1899

chapel—nuns, doctors, nurses, matron, and patients—received the Sacrament, kneeling in rows beside one another upon the pavement, without thought, for this one morning, of official rank and social differences.

Afterwards came the nurses' breakfast, nearly three-quarters of an hour late. But are not crackling sausages, fried bacon and eggs, and hot cakes worth waiting for, when you get them only once a year? We breakfasted gorgeously that morning, and so did the patients, for the sisters of the various wards dealt out luxuries to all with a liberal hand.

"I know they'll all be worse at the end of the day," says Sister S——, with a twinkle of her merry, grey eye—"for every locker except the typhoids' is full of cake and sweets and oranges; and I'm afraid to think of what the visitors will bring on the top of that. No, Eighteen, you needn't be afraid, you shall have your bacon all the same. It's Christmas Day."

"Begorra, thin, lave me plinty, Sisther; sure it has to last me to next Christmas Eve!" observes Eighteen, peering under the bandages that wrap his head.

There is an air of determined gaiety about the wards this morning; the decorations are marvellously elaborate, and generally very pretty, although the conventual fancy for paper flowers has been allowed to run riot somewhat here and there. Paddy, the ward-boy of St. K——'s male surgical ward, has developed a sudden and unexpected access of intelligence. Outside his work, which he knows surprisingly well, Paddy is a trifle simple in mind; or, as his own countrymen say, he is "not that wise." But this morning Paddy has appeared with some Christmas roses and a fresh trail of ivy, gathered off the convent garden wall, to decorate the only unadorned gas-bracket, and insists on doing it all himself, with considerable artistic taste.

St. K——'s ward begins to giggle when he is perched on the ladder, half way up, twisting and tying; for Surgeon O'P—— (of whom Paddy stands in terror since last Wednesday, when he carelessly threw away a grisly bottled curiosity that the doctor wished to keep) has come noiselessly into the ward, and slipped on tiptoe to the foot of the ladder, where he stands glaring up at the luckless Paddy, caught at last. Surgeon O'P—— is in reality the kindest of men, but he doesn't look it, and Paddy certainly does not believe he is, but fears him like the foul fiend himself.

"Now, Sister, that'll put a taste of quality into the place. Did ye ever see the like? and amn't I the boy can . . . . Jesus-Mary-an'-Joseph-the-Cross-of-Christ-be-about-us!"

Paddy has scuttled like a squirrel to the top of the long ladder, ejaculating piety that sounds more like profanity, with the accent he gives it. Surgeon O'P—— is standing at the foot of the ladder, motionless and fierce, holding a glittering bistoury in his hand.

The ward is roaring with laughter, but Paddy thinks discretion the better part of valour, and flatly refuses to come down until Surgeon O'P——, laughing as much as anyone, has finished his round and gone away.

"On Christmas morning, too—the black-hearted old naygur!" he says pathetically, as he creeps back to terra firma at last.

St. Rose's ward, the women's surgical, is a good deal quieter. All the patients who could go home for Christmas have gone, and those who cannot, or who have no home to go to, are gay more by force of will than conviction. A woman takes much less kindly to hospital than a man, in any case; but an Irishwoman in hospital at Christmas-tide, with the "blessed childher" alone at home——!

Crowds of well-dressed visitors belonging to the world of philanthropy come in during the morning, to walk down the wards, give presents, and talk kindly, patronising talk. The patients receive them with the innate politeness of the Celt; but when their own visitors come in later on it is another story. There is much more kissing and hugging than usual to-day, a little more crying, and a few more parcels—especially the (invisible) flat parcels which are Anathema Maranatha to the watchful sisters, and must be looked for as soon as the friends have gone.

Of course there is turkey and plum-pudding for the atients' dinner; but the cakes and sweets of the morning are telling their tale, and appetites are small. When the nurses' dinner—which is also one with many frills—is over, and half the nursing staff have gone off duty—Sunday hours being kept—a stillness falls upon the wards, broken happily into by a party of lady and gentleman singers, who have come to cheer the afternoon, and not a minute too soon.

After tea, when the wards are beginning to settle down for the night, those patients who are too ill to dress and go to the nurses' concert in the theatre, drop the mask altogether, and in dim corners of the wards there is subdued sobbing here and there. The Irish peasant is above all things home-loving and family-worshipping, and the joviality of an English hospital at Christmastide is a thing impossible to him or her.

Among nurses and doctors there is freer association than on ordinary days, also a good deal of quiet flirtation; while even a trifle of romping among the ivy garlands of the hall is winked at by the nuns—if a nun can wink. But after all, and through all, and at the end of all, there is an Irish wail in every one of our hearts—just the familiar wail of the child brought into hospital for the first time—"I want to go home!"

And when the concert is over, and the lights out, and we all gather in the hall ready to cross the road once more, a nurse from the far-away South whispers to a student from the distant West—"Aren't you glad it's all over." And the student says, "Most confoundedly glad!"

### SWEET CHARITY'S GUIDE TO CHRISTMAS GIVERS.

### GENERAL CHARITIES—*Concluded.*

this society obtained 7,900 convictions against cruel persons in different parts of the country. These proceedings involved great cost to the society, and an earnest appeal is made for increased financial support to prevent a curtailment of operations. Secretary, Mr. John Colam, 105 Jermyn Street, London, S.W.

**Shipwrecked Fishermen and Mariners' Royal Benevolent Society,** 26 Suffolk Street, Pall Mall East, S.W.—The work done by this society includes the granting of relief to shipwrecked persons, to widows and orphans of members and others, to destitute seamen, &c.; grants towards meeting loss of clothes or boats, assistance in old age or in case of infirmity. Over £21,000 was expended in relief in 1899 to 10,648 persons. Secretary, Mr. Gerald E. Maude.

## Figures and Tables

**Figure f1:**